# Ammonium Glycyrrhizinate-Reinforced Dual-Network Poly(Thioctic Acid)-Based Hydrogel Dressing with Robust Wet Adhesion, Antibacterial Activity and Oxidative Stress Regulation

**DOI:** 10.3390/ma19112388

**Published:** 2026-06-03

**Authors:** Ziming Cheng, Zhiyuan Zhang, Huanfu Lu, Jiawei Zhang, Yang Yuan, Fangzheng Yu, Chen Wang, Jiale He, Zheng Zhao

**Affiliations:** 1 State Key Laboratory of Advanced Technology for Materials Synthesis and Processing, Wuhan University of Technology, Wuhan 430070, China; chengziming0814@gmail.com (Z.C.); 19863751715@163.com (Z.Z.); zjw9801@126.com (J.Z.); 348318@whut.edu.cn (Y.Y.); 348317@whut.edu.cn (F.Y.); wangchen__123@163.com (C.W.); hjl2024305118@163.com (J.H.); 2 Sanya Science and Education Innovation Park, Wuhan University of Technology, Sanya 572000, China; 3 Hainan Finsen Medical Devices Co., Ltd., Haikou 570100, China; 13098956303@139.com

**Keywords:** dual-network, wet tissue adhesion, antioxidant activity, antibacterial activity

## Abstract

Developing hydrogel dressings that simultaneously achieve robust wet tissue adhesion, mechanical stability, antibacterial activity, and oxidative stress regulation remains challenging. In this study, a dual-network poly (thioctic acid)/ammonium glycyrrhizinate (PTA/AG) hydrogel was developed through thermally induced ring-opening polymerization (ROP) of TA and sodium thioctate (TA-Na) to form a primary network, followed by the formation of an AG-driven secondary network during cooling. TA-Na improved the aqueous processability of TA, while the AG secondary network reinforced the stability of the PTA primary network. The resulting hydrogel exhibited a crossover strain of 454% and a wet adhesion strength of up to 16.37 kPa on porcine skin. In addition, the hydrogel showed strong antibacterial activity against *S. aureus* (>99%), high cytocompatibility (>95% cell viability), and effective free-radical-scavenging activity (>77% scavenging of both DPPH and ABTS radicals). Notably, the hydrogel exhibited effective intracellular antioxidant activity, reducing ROS levels to near those of the control group and increasing SOD activity by approximately 13-fold and the GSH/GSSG ratio by 97.83% relative to the H_2_O_2_ group. Overall, the PTA/AG hydrogel is a promising candidate for multifunctional wound dressing applications.

## 1. Introduction

In recent years, hydrogel dressings have attracted increasing attention in wound management because their hydrated three-dimensional networks help maintain a moist microenvironment and enable close conformity to irregular tissue surfaces [1,2]. However, achieving robust wet tissue adhesion while simultaneously maintaining mechanical integrity and therapeutic bioactivity remains challenging [3,4]. First, many hydrogel dressings exhibit insufficient mechanical strength. Therefore, they are prone to deformation, interfacial failure, or detachment under dynamic wound conditions, thereby hindering stable adhesion [5]. Second, wounds are typically found in wet environments with exudates. The hydration layer formed at the wet interface weakens hydrogel–tissue interactions and limits stable adhesion to the wound surface [6]. Finally, insufficient antibacterial and antioxidant activities further limit the ability of hydrogel dressings to provide a favorable microenvironment for wound healing [3,5,7].

Thioctic acid (TA) has been regarded as a promising building block for hydrogel dressings because of its favorable biocompatibility, antioxidant activity, and adhesive properties [8,9]. TA can undergo thermally induced ring-opening polymerization (ROP) through its 1,2-dithiolane ring to form dynamic poly (thioctic acid) PTA. The multiple potential adhesive sites along the PTA backbone make it a suitable candidate for use as an adhesive matrix. Meanwhile, the 1,2-dithiolane group can facilitate hydrophobic exclusion of interfacial water, which is beneficial for wet adhesion. However, the hydrophobic 1,2-dithiolane ring and alkyl chain of TA result in limited water solubility, which hinders hydrogel formation under wet conditions [10]. Compared with TA, sodium thioctate (TA-Na) exhibits higher water solubility because of its carboxylate salt form. Importantly, TA-Na retains the characteristic 1,2-dithiolane ring structure of TA, allowing it to participate in similar dynamic chemical processes [11]. In addition, TA-Na may reduce the hydrophobic aggregation of TA molecules and promote their more homogeneous distribution in wet conditions [12]. Therefore, TA-Na was introduced to improve the aqueous processability and compositional homogeneity of TA during hydrogel formation.

Although processability and network uniformity are beneficial, they do not directly address the challenge of treating infected wounds, which requires additional antibacterial functionality. PTA-based hydrogels exhibit limited antibacterial efficacy, which is insufficient to meet the requirements for infected wound treatment [13]. In contrast, ammonium glycyrrhizinate (AG), a triterpenoid saponin derived from licorice, has been reported to effectively inhibit specific pathogens such as *S. aureus* by disrupting bacterial biofilms. In addition, AG has shown considerable potential for antioxidant and cytoprotective applications [14]. Current studies suggest that the reactive oxygen species (ROS)-scavenging activity of AG is not solely dependent on direct chemical reduction; it also involves the multi-targeted regulation of endogenous antioxidant networks [15,16]. Therefore, AG may provide sustained and biologically relevant protection by directly scavenging ROS and enhancing endogenous antioxidant defenses. However, self-assembled AG physical hydrogels exhibit pronounced mechanical brittleness, limiting their ability to withstand interfacial shear stress in biological tissues. Thus, AG is a suitable candidate for constructing a secondary biofunctional network within PTA-based hydrogel.

**Scheme 1:**
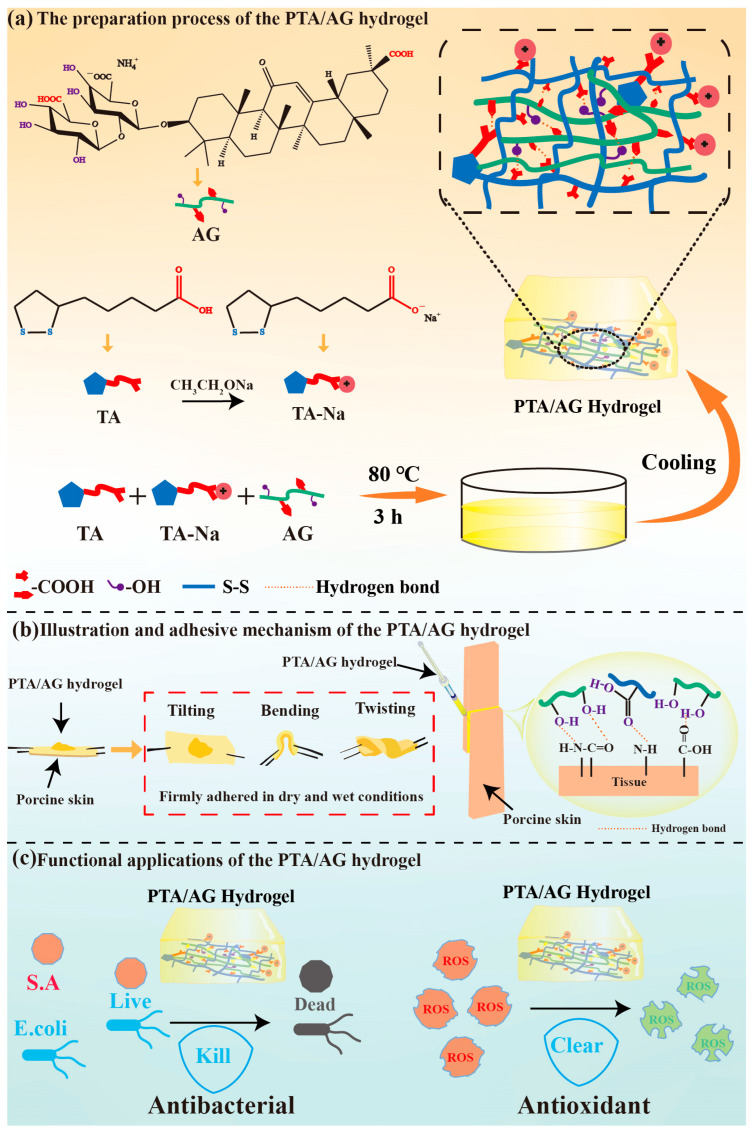
(**a**) Illustration of the preparation process of the PTA/AG hydrogel and (**b**) illustration and mechanism of PTA/AG hydrogel adhesion on porcine skin. (**c**) Functional applications of the PTA/AG hydrogel.

In this work, a PTA/AG hydrogel was designed by combining a PTA primary network with an AG-derived supramolecular secondary network. During the heating process, TA and TA-Na underwent ROP to form a PTA primary network, whereas AG self-assembled into a secondary network during the subsequent cooling process, thereby yielding a PTA/AG dual-network hydrogel (Scheme 1a). TA-Na was introduced to improve the aqueous processability of TA, whereas AG served as both a bioactive molecule and a secondary-network component to reinforce the hydrogel network through self-assembly and non-covalent interactions (Scheme 1b). Furthermore, the physical and chemical properties, wet adhesion, antioxidant and antibacterial abilities, and biocompatibility of the PTA/AG hydrogels were systematically investigated (Scheme 1c).

## 2. Materials and Methods

### 2.1. Materials

Thioctic acid (TA) was purchased from Maclean Biochemical Co., Ltd. (Shanghai, China), and ammonium glycyrrhizinate (AG) and sodium ethoxide were purchased from Aladdin Biochemical Co., Ltd. (Shanghai, China). RPMI 1640 medium was purchased from Gibco Life Sciences (Grand Island, NY 14072-2028, USA) [17,18], and fetal bovine serum (FBS), penicillin–streptomycin (PS), calcium yellow green AM staining, Cell Counting Kit-8 (CCK-8), and phosphate-buffered saline (PBS) were provided by Beyotime Biotechnology (Shanghai, China).

### 2.2. Preparation of Sodium Thioctanoate (TA-Na)

Firstly, TA-Na was prepared as the raw material for synthesizing hydrogel. A total of 1.7 g of sodium ethoxide and 5.15 g of TA were separately dissolved in 50 mL of anhydrous ethanol to obtain the sodium ethoxide solution and TA solution, respectively. After complete dissolution, the TA solution was added dropwise into the sodium ethoxide solution using a rubber-tipped burette. A large amount of flocculent precipitate was observed during the reaction. The mixture was stirred for 12 h. After that, the solvent was removed by rotary evaporation, and TA-Na was obtained.

### 2.3. Preparation of Poly(Thioctic Acid) (PTA) Hydrogels

The PTA hydrogel was prepared under ambient atmosphere. A total of 1.67 g of TA-Na was dissolved in 5 mL of deionized water in a round-bottomed flask, followed by the addition of 0.83 g of TA powder to adjust the pH of the precursor solution to approximately 7.5. The mixture was heated and stirred at 80 °C for 3 h. This formulation corresponded to concentrations of 334 mg/mL TA-Na and 166 mg/mL TA with an approximate molar ratio of TA:TA-Na of 1:1.82. TA and TA-Na underwent thermally induced ring-opening polymerization at 80 °C through dynamic disulfide bond exchange to form the hydrogel [9].

### 2.4. Preparation of Ammonium Glycyrrhizinate (AG) Hydrogels

The AG hydrogel was prepared under ambient atmosphere. A total of 0.5 g of AG powder was dissolved in 5 mL of deionized water, followed by heating and stirring at 80 °C for 3 h. This formulation corresponded to an AG concentration of 100 mg/mL. Upon cooling to room temperature, the AG molecules underwent self-assembly [19], resulting in the formation of a physically crosslinked hydrogel.

### 2.5. Preparation of PTA/AG Hydrogels

The PTA/AG hydrogel was prepared under ambient atmosphere. Briefly, 1.67 g of TA-Na was dissolved in 5 mL of deionized water in a round-bottomed flask, followed by the addition of 0.83 g of TA powder to adjust the pH of the precursor solution to approximately 7.5. Subsequently, 0.2 g of ammonium glycyrrhizinate (AG) powder was added. The mixture was heated and stirred at 80 °C for 1, 2, or 3 h. This formulation corresponded to final concentrations of 334 mg/mL TA-Na, 166 mg/mL TA, and 40 mg/mL AG, with an approximate molar ratio of TA:TA-Na:AG of 1:1.82:0.059. The gelation behavior was evaluated using the vial-inversion/tilting method. The PTA/AG hydrogel heated for 3 h was selected for subsequent experiments because it showed stable gelation without obvious fluidity after cooling. To confirm reproducibility, the PTA/AG hydrogel was prepared in three independent batches under the same conditions, and stable gel formation was consistently observed.

### 2.6. Gel Fraction Test

The gel fraction of the PTA/AG hydrogel was determined by a gravimetric method [20]. Briefly, the freshly prepared hydrogel samples were freeze-dried to a constant weight, and the initial dry weight was recorded as $W_{0}$. The dried hydrogel samples were then immersed in deionized water at 37 °C for 24 h to remove unreacted monomers, soluble oligomers, and weakly bound components. After immersion, the samples were gently rinsed with deionized water and freeze-dried again to a constant weight. The remaining dry weight was recorded as $W_{d}$. Tests were performed in three independent batches, and the results were expressed as mean ± standard deviation. The gel fraction was calculated according to the following equation:

(1) $$Gel\;fraction\;\left(\%\right)=\frac{W_{d}}{W_{0}}\times 100\%$$

### 2.7. Fourier Transform Infrared (FT-IR) Spectroscopy

FT-IR spectra of AG, TA, TA-Na, and PTA/AG hydrogel samples were recorded using a Nexus FT-IR spectrometer (Thermo Nicolet, Waltham, MA, USA). Before measurement, the hydrogel samples were freeze-dried to remove water. The dried samples were mixed with potassium bromide (KBr) powder at an appropriate ratio and thoroughly ground in an agate mortar to obtain a homogeneous fine powder. The mixture was then pressed into transparent pellets for FT-IR analysis. Spectra were collected over the range of 4000–400 cm^−1^ with a resolution of 4 cm^−1^, and 32 scans were accumulated for each spectrum. Each sample was measured three times to ensure reproducibility.

### 2.8. Scanning Electron Microscopy (SEM) Test

The freeze-dried hydrogel samples were mounted on conductive adhesive tape and sputter-coated with a thin conductive layer. SEM images were obtained using a scanning electron microscope (JSM-IT200, JEOL, Tokyo, Japan) at 5.0 kV and 60× magnification. The morphology of different samples was compared using images collected under 60× magnification and scale bar = 200 μm.

### 2.9. Rheological Testing

The rheological behavior of hydrogels was evaluated by a rotational rheometer (Kinexus pro+). Strain amplitude sweeps were performed with a continuous strain range of 0.1–1000% at a fixed frequency of 1 rad/s to determine the linear viscoelasticity region and evaluate the stability and critical strain points of AG, PTA and PTA/AG hydrogels. Oscillation frequency sweeps were used to assess the rheological properties of the PTA/AG hydrogels at 25 °C using a rotational rheometer with 20 mm parallel plate geometry and a 1000 μm gap. Oscillation frequency sweeps (from 0.1 to 10 Hz) were conducted to assess the stiffness of the hydrogel fixed at 1% strain.

### 2.10. Compressive Stress Test

Cylindrical hydrogel samples (d = 20 mm, h = 15 mm) were subjected to compression testing with a 50 N sensor (compression rate: 10 mm/min). The stress at 80% strain was taken as the compressive strength of the hydrogel [21]. The slope of the strain–stress curve, from 10% to 15% strain, was used to determine the Young’s compressive modulus [22].

### 2.11. Adhesion Performance Test

The adhesion of the PTA/AG hydrogel to the surface of porcine skin was observed by tilting, bending, and twisting the porcine skin in a glass jar filled with deionized water and observing the adhesion of the hydrogel to the surface of the porcine skin. An underwater adhesion test was also conducted, in which the PTA/AG hydrogel was placed in deionized water for 6 h, and its adhesion was tested under a mechanical testing machine.

The adhesion properties of the hydrogels were quantitatively evaluated by lap-shear experiments. Fresh porcine skin measuring 25 mm × 75 mm was coated with 25 mm × 40 mm of PTA/AG hydrogel. A universal testing machine was then used to apply tensile force to the samples at a strain rate of 5 mm/min until the two skin samples were completely separated. Three parallel tests were performed for each experimental group to ensure reliability. The adhesion strength was calculated from the results obtained. The adhesion strength was determined as follows:

(2) $$\mathrm{Adhesive}\;\mathrm{strength}\;(kPa)=\frac{F}{S}$$

where *F* is the highest test force and *S* is the area intersected by the lap shear.

### 2.12. In Vitro Free-Radical-Scavenging Experiment

In the experiment, 1,1-diphenyl-2-trinitrophenylhydrazine (DPPH) and 2,2′-azino-bis-3-ethylbenzthiazoline-6-sulphonic acid (ABTS) were chosen as the free radical for the antioxidant test. Firstly, the ABTS solution was prepared by weighing 0.1015 g of ABTS powder in a flask and adding 25 mL of ethanol to dissolve it. Weigh 0.0148 g of ammonium persulfate in 25 mL of ethanol and mix the two solutions overnight to obtain the ABTS solution. The OD value was measured by heating 0.5 mL of the day-old material with 5 mL of ABTS solution at 37 °C for 12 h. A total of 0.5 mL of deionized water was added to 5 mL of ABTS as a control for the experiment. The od value was measured at fixed intervals at 517 nm (DPPH) and 734 nm (ABTS) using a UV-vis spectrophotometer and finally calculated by the following formula (data are mean ± SD, *n* = 3):

(3) $$\mathrm{Scavenging}\;\mathrm{activity}\;\left(\%\right)=\frac{A_{0}-A_{1}}{A_{0}}\times 100\%$$

where *A*_0_ denotes the OD value of the control test and *A*_1_ denotes the OD value of the hydrogel group.

### 2.13. In Vitro Antibacterial Assessment

The antibacterial activities of PTA, AG, and PTA/AG hydrogels against Gram-positive *S. aureus* and Gram-negative *E. coli* were evaluated using the spread plate method. For each sample, 100 μL of hydrogel was added to a 48-well plate and sterilized under UV light for 12 h. Then, 1 mL of activated bacterial suspension with an initial concentration of approximately 1 × 10^5^ CFU/mL was added to each well. The blank control group and hydrogel-treated groups were co-cultured at 37 °C and 120 rpm for 24 h. After incubation, 100 μL of bacterial suspension from each group was collected and serially diluted with PBS to achieve a 10^5^-fold dilution. Subsequently, 100 μL of the diluted suspension was evenly spread onto agar plates and incubated overnight at 37 °C. The bacteria were photographed and counted. Then the bactericidal rate was calculated by the following formula:

(4) $$\mathrm{Antibacterial}\;\mathrm{ratio}\;(\%)=1-\frac{A}{A_{0}}\times 100\%$$

where *A* is the number of bacteria in the material group and *A*_0_ is the number of bacteria in the control group.

### 2.14. In Vitro Cytocompatibility Assessment of Hydrogels

L929 fibroblasts were utilized to quantitatively assess the cytocompatibility of hydrogels using the CCK-8 reagent. Briefly, L929 cells (Wuhan Servicebio Technology Co., Ltd., Wuhan, China) [18] were seeded into 96-well plates at a density of 5000 cells per well and cultured in a medium comprising 1640 medium supplemented with 10% fetal bovine serum and 1% penicillin/streptomycin at 37 °C in a 5% CO_2_ atmosphere. Concurrently, the materials were immersed in 1640 medium supplemented with 1% penicillin/streptomycin and 10% fetal bovine serum and left for 24 h. The soaked culture broth was filtered through a biofiltration membrane to obtain hydrogel extracts. After one day, the culture medium was aspirated, and the PTA/AG hydrogel extracts were added for co-cultivation over 1 and 3 days. At the specified time points, the medium was aspirated, and 100 µL of CCK-8 reagent was added, followed by incubation for 2 h at 37 °C in a 5% CO_2_ environment. After the incubation period, the absorbance at 450 nm was measured using an enzyme meter. The cell viability was calculated using the following formula:

(5) $$Cell\;viability\;\left(\%\right)=\frac{A_{T}}{A_{C}}\times 100\%$$

where $A_{T}$ and $A_{C}$ represent the absorbance values of the test and control groups, respectively.

To demonstrate the cytocompatibility of hydrogels more closely, we utilized cell live/dead assays to stain cells using the Calcein-AM/PI staining, and observed the state of cells co-cultured in hydrogels under a fluorescence microscope.

### 2.15. Assessment of the Reactive Oxygen Species (ROS) Scavenging Efficiency of PTA/AG Hydrogel

An oxidative stress model was established using H2O2 to evaluate the protective effect of the PTA/AG hydrogel on human umbilical vein endothelial cells (HUVECs, Wuhan Servicebio Technology Co., Ltd., Wuhan, China) [23] under oxidative stress conditions. Sterile water was used as the control. HUVECs (1 × 10^4^ cells/well) were seeded into 48-well plates and cultured for 24 h. The cells were then treated with 100 μM H_2_O_2_ for 30 min, followed by washing three times with PBS. Subsequently, 1 mL of PTA/AG hydrogel extract was added and incubated with the cells for 30 min. Intracellular reactive oxygen species (ROS) levels were detected using 2′,7′-dichlorodihydrofluorescein diacetate (DCFH-DA) under dark conditions and observed with a fluorescent inverted microscope (Olympus IX71, Tokyo, Japan).

To further assess the antioxidant capacity of the PTA/AG hydrogel, intracellular superoxide dismutase (SOD) activity and glutathione/oxidized glutathione disulfide (GSH/GSSG) levels were measured using a total SOD assay kit with WST-8 and a GSH/GSSG assay kit (Beyotime Biotech, Shanghai, China), respectively. Briefly, HUVECs were cultured for 24 h and exposed to 100 μM H_2_O_2_ to induce oxidative stress, followed by incubation with PTA/AG hydrogel extract for 30 min. After washing with pre-cooled PBS, the cells were collected and processed according to the manufacturers’ instructions. For SOD activity, the absorbance was measured at 450 nm. For GSH/GSSG determination, the absorbance was recorded at 412 nm.

### 2.16. Degradation Properties of Hydrogel

The initial dry weight of the PTA/AG hydrogel was recorded as W_1_. It was then immersed in PBS and incubated in a constant temperature air bath at 37 °C with a shaking speed of 100 rpm. At predetermined time intervals, hydrogels were retrieved, and surface moisture was carefully removed using filter paper. Following complete drying until constant weight, the dry mass was measured and designated as W_2_. The degradation rate of the PTA/AG hydrogel was calculated using the following formula [24,25,26]:

(6) $$Dagradation\;rate\;(\%)=\frac{W_{1}-W_{2}}{W_{1}}\times 100\%$$

### 2.17. Statistical Analysis

Each experiment was performed 3 times (*n* = 3). All experiments in this study were conducted with three or more parallel samples, and the data were expressed as mean ± standard deviation. The data were obtained by one-way analysis of variance (**** *p* < 0.0001, *** *p* < 0.001, ** *p* < 0.01, * *p* < 0.05, ns *p* > 0.05).

## 3. Results and Discussion

### 3.1. Preparation and Characterization of PTA/AG Hydrogel

**Figure 1:**
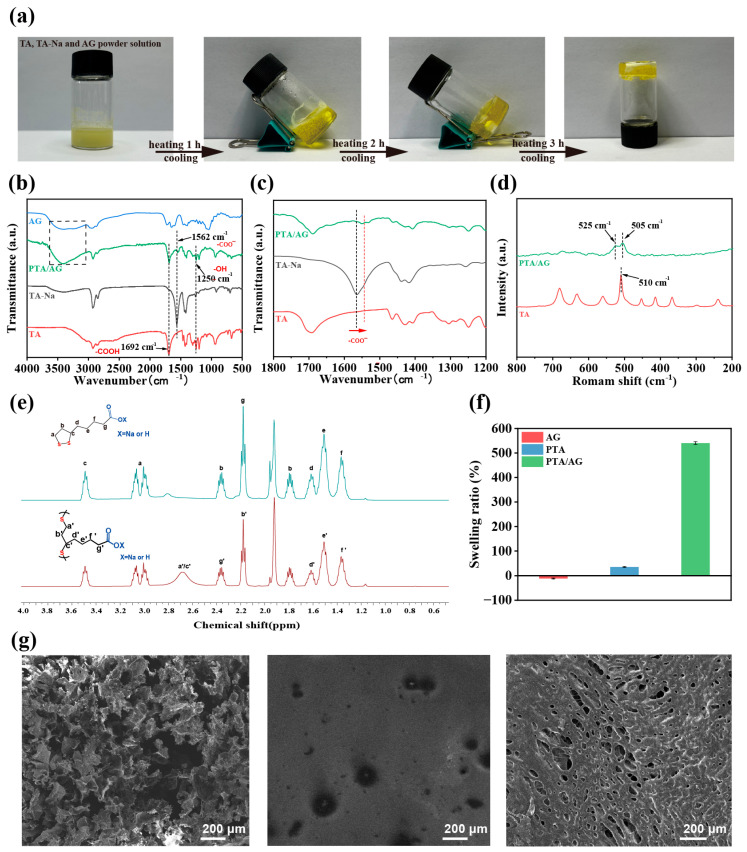
(**a**) Preparation of PTA/AG hydrogel and its gelation under different heating times. (**b**) The FT-IR spectra of TA, TA-Na, AG powders and PTA/AG hydrogels in the wavenumber range of 4000 cm^−1^ to 500 cm^−1^ and (**c**) 1800 cm^−1^ to 1200 cm^−1^. (**d**) Raman spectra of the TA and the PTA/AG hydrogel. (**e**) ^1^H NMR spectrum of TA and the PTA/AG hydrogel. (**f**) Swelling ratios of the AG, PTA and PTA/AG hydrogels. (**g**) From left to right are the electron microscope scanning observation images of AG, PTA and PTA/AG hydrogels in sequence (*n* = 3).

The gelation performance of PTA/AG hydrogels under different heating durations was assessed. As shown in Figure 1a, heating durations led to different degrees of polymerization in the PTA network, thereby influencing the final morphology of the hydrogel. The TA/AG mixture heated for 1 h was cooled and tilted, and the resultant gel retained fluidity. In contrast, after 2 h of heating, the gel exhibited a reduced flow tendency upon tilting. After 3 h of heating, no fluidity was observed after cooling, indicating the successful formation of the PTA/AG hydrogel. The gel fraction of the PTA/AG hydrogel was 71.34 ± 0.70%. These results indicated that prolonged heating enhances the polymerization degree of the PTA network. TA and TA-Na may undergo thermally induced ring-opening polymerization through dynamic disulfide bond exchange, thereby forming a PTA primary network [27,28]. During cooling, AG could self-assemble into a secondary physical network [29], which may interact with the PTA primary network mainly through physical interactions, including topological entanglement, hydrogen bonding, hydrophobic association, and other non-covalent interactions, thereby reinforcing the hydrogel network.

The formation mechanism of the PTA/AG hydrogel was investigated by Fourier transform infrared spectroscopy (FTIR) (Figure 1b,c). First, in the TA-Na structure, the –OH absorption peak of the carboxyl group at 1250 cm^−1^ almost disappeared, while the –C=O absorption peak shifted from 1692 cm^−1^ to 1562 cm^−1^ (Figure 1b), indicating that most carboxyl groups in TA-Na were deprotonated and that the original hydrogen-bonding interactions were disrupted [27]. In contrast, the reappearance of the –OH absorption peak at 1250 cm^−1^ in the PTA/AG hydrogel indicated the coexistence of TA and TA-Na in the network (Figure 1b). Moreover, compared with TA-Na, the carboxylate-related –C=O absorption peak in the PTA/AG hydrogel exhibited an obvious red shift (Figure 1c), suggesting the formation of strong hydrogen-bonding interactions between the –COOH groups in TA and the –COO^−^ groups in TA-Na [27]. The broad absorption band observed at 3600–3200 cm^−1^ in the PTA/AG hydrogel corresponded to the characteristic –OH absorption of AG (Figure 1b) [30]. The result indicated that AG was successfully incorporated into the hydrogel network. As shown in Figure 1d, the Raman characteristic peak of the five-membered-ring disulfide bond at 510 cm^−1^ splits into two peaks at 525 cm^−1^ and 505 cm^−1^. This result indicated that ROP of TA and TA-Na in the PTA/AG hydrogel was successfully achieved at 80 °C [31]. Consistently, as shown in Figure 1e, the ^1^H NMR spectrum further confirmed the formation of PTA, as evidenced by the peak at 2.68 ppm in the PTA/AG hydrogel, which suggested the transformation of TA into PTA [26].

In addition, the swelling behavior of AG, PTA, and PTA/AG hydrogels was evaluated by immersing the pre-weighed lyophilized hydrogels in 100 mL of PBS. As shown in Figure 1f, the swelling ratios of the AG, PTA, and PTA/AG hydrogels were −11.09 ± 1.86%, 35.96 ± 1.83%, and 540.43 ± 5.61%, respectively. The results showed that the PTA/AG hydrogel exhibited a significantly higher swelling ratio than the AG and PTA hydrogels, which may be attributed to its more developed porous network structure. This porous architecture could provide more space for water uptake, thereby improving the swelling capacity of the hydrogel. The negative swelling ratio of AG hydrogel may be ascribed to the partial dissolution and diffusion of AG molecules from the hydrogel network into the surrounding medium during immersion, resulting in a slight mass loss.

Finally, the lyophilized samples of AG, PTA, and PTA/AG hydrogels were characterized by scanning electron microscopy (SEM) to systematically investigate their pore structures. As shown in Figure 1g, SEM images showed that the AG hydrogel exhibited a relatively loose and porous structure, whereas the PTA hydrogel presented a much denser morphology with fewer visible pores, indicating a lower porosity. In contrast, the PTA/AG hydrogel displayed a more uniform and well-developed porous network with interconnected micropores. This difference may be attributed to the reinforcement of the PTA primary network by the AG self-assembled secondary network. Specifically, AG contributed to the formation of a more open network, whereas PTA provided structural support to stabilize the porous architecture, thereby promoting a more homogeneous three-dimensional porous structure in the PTA/AG hydrogel.

### 3.2. Mechanical Characterization of Hydrogels

**Figure 2:**
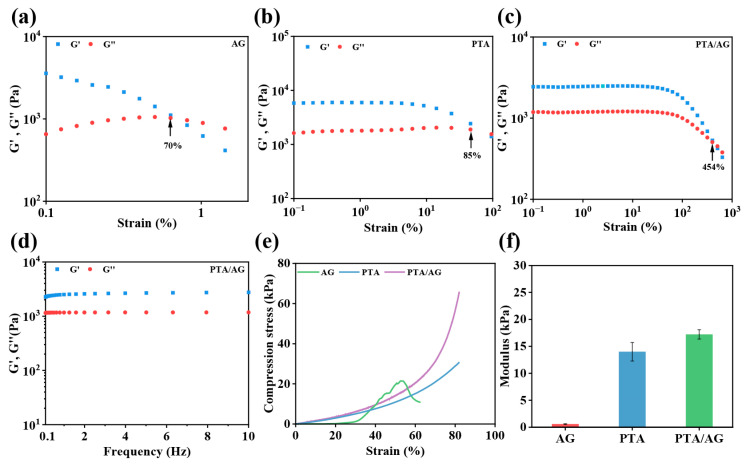
Strain-sweep rheological curves of (**a**) AG, (**b**) PTA and (**c**) PTA/AG hydrogel. (**d**) Frequency sweep curve of PTA/AG hydrogel (fixed at 1% strain). (**e**) Compression stress curve of the hydrogels. (**f**) Compressive Young’s modulus of AG, PTA and PTA/AG hydrogels (*n* = 3).

Good rheological properties provide hydrogels with high deformation resistance and structural stability, allowing them to maintain integrity under friction and other external stresses in dynamic physiological environments, thereby reducing the risk of dressing failure [5,32]. As shown in Figure 2a–c, all hydrogel groups exhibited gel-like behavior at low strain, with G′ consistently higher than G″, indicating an elastic-dominant response under small deformation. For the AG hydrogel, the crossover point between G′ and G″ appeared at approximately 70% strain, suggesting that the AG network yielded at a relatively low deformation level. When the applied strain exceeded this critical value, G″ became higher than G′, indicating disruption of the hydrogel network and a transition from solid-like to liquid-like behavior. By comparison, the PTA hydrogel showed a slightly higher crossover strain of approximately 85%, indicating better deformation tolerance than the AG hydrogel. Notably, the PTA/AG hydrogel exhibited the highest crossover strain, reaching 454%, which demonstrated a markedly enhanced resistance to deformation. As shown in Figure 2d, the PTA/AG hydrogel maintained G′ higher than G″ over the tested frequency range (0.1–10 Hz), indicating a stable elastic-dominant hydrogel network. This result was likely attributed to the effect of the dual-network structure, which promoted stronger intermolecular interactions and more efficient stress dissipation within the hydrogel network.

Compression tests were performed to further evaluate the mechanical stability of the PTA/AG hydrogel. As shown in Figure 2e, the PTA/AG hydrogel exhibited a compressive strength of 67 kPa, which was higher than that of the PTA hydrogel (52 kPa) and AG hydrogel (21 kPa). Its compressive Young’s modulus reached 17 kPa (Figure 2f), which was close to that of human skin (1–100 kPa), suggesting good mechanical compatibility with native human skin [33]. These results may be attributed to the stable dual-network structure of the PTA/AG hydrogel, in which the PTA primary network provided the basic elastic framework, whereas the AG self-assembled secondary network further reinforced the hydrogel through hydrogen bonding, hydrophobic association, and physical entanglement. Overall, the rheological and compression stress results demonstrate the good mechanical robustness of the PTA/AG hydrogel.

### 3.3. Adhesive Properties of PTA/AG Hydrogel

**Figure 3:**
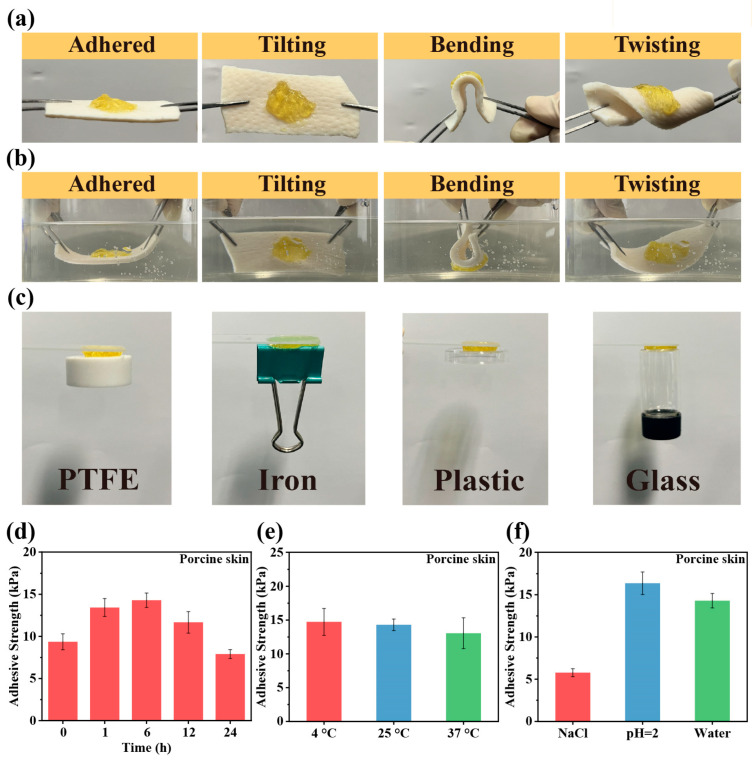
(**a**) The PTA/AG hydrogel adhered to porcine skin under dry states withstand tilting, bending, and twisting. (**b**) The PTA/AG hydrogel adhered to porcine skin under wet states withstand bending and twisting. (**c**) Adhesion of the PTA/AG hydrogel to different substrates. (**d**) The wet adhesive strength of the PTA/AG hydrogel to porcine skin after 0, 1, 6, 12, and 24 h in water. (**e**) The wet adhesive strength of the PTA/AG hydrogel adhered to porcine skin and stored at 4, 25, and 37 °C after 6 h. (**f**) The wet adhesion strength of the PTA/AG hydrogel to porcine skin in NaCl (1 mol/L) solution, water, and acidic solution (pH = 2) after 6 h (*n* = 3).

Hydrogel dressings with strong skin adhesion are more suitable for wound healing [34]. As shown in Figure 3a,b, the PTA/AG hydrogel can firmly adhere to porcine skin in both dry and wet conditions. The hydrogel on porcine skin withstood tilting, bending, and twisting without detachment. These results indicated that the PTA/AG hydrogel exhibited good skin adhesion and deformation flexibility. The favorable adhesive performance of the PTA/AG hydrogel may be attributed to the synergistic contribution of strong interfacial interactions and its robust dual-network structure. On the one hand, the PTA/AG hydrogel contains abundant carboxyl-, hydroxyl-, and disulfide-containing groups, which can establish multiple physical interactions with the tissue surface, including hydrogen bonding, hydrophobic interactions, and intermolecular chain entanglement. These functional groups facilitate intimate interfacial contact with porcine skin, whereas the hydrophobic moieties help reduce the interference of interfacial water, thereby contributing to stable adhesion under both dry and wet conditions. On the other hand, the dual-network structure composed of the PTA primary network and AG secondary network provides sufficient cohesion and deformation tolerance, enabling the hydrogel to remain attached during bending, twisting, and tilting without detachment.

Figure 3c shows the immediate adhesion properties of the PTA/AG hydrogel to different materials. Various substrates (such as PTFE, iron, plastic and glass) could also be glued to a glass rod by using the PTA/AG hydrogel. Furthermore, the adhesive strength of the PTA/AG hydrogel was measured using a lap-shear assay. As shown in Figure 3d, the adhesive strengths of the PTA/AG hydrogel adhered to porcine skin after 0, 1, 6, 12 and 24 h in water were 9.36 ± 0.95, 13.43 ± 1.07, 14.30 ± 0.85, 11.67 ± 1.27 and 7.90 ± 0.52 kPa, respectively. As the immersion time increased to 6 h, the adhesive strength of the PTA/AG hydrogel gradually increased and reached its maximum value. However, after 24 h of adhesion to porcine skin in water, the adhesive strength decreased to 7.90 ± 0.52 kPa. This result showed that the PTA/AG hydrogel had the best adhesion performance in water at 6 h. Therefore, a hydrogel adhesion time of 6 h was used for the next adhesive experiments. The time-dependent wet adhesion behavior in water suggested a dynamic interfacial adaptation process of the PTA/AG hydrogel. From 0 to 6 h, the adhesive strength gradually increased. This increase may be attributed to progressive interfacial adaptation, during which the carboxyl and hydroxyl groups of the PTA/AG hydrogel gradually formed hydrogen bonds and physical entanglements with amino- and carboxyl-containing components on the porcine skin surface, while the disulfide-containing hydrophobic moieties helped exclude the interfacial hydration layer, thereby enhancing wet adhesion. In addition, short-term immersion may facilitate further stabilization of physical interactions within the PTA/AG hydrogel network, leading to enhanced adhesion. However, after prolonged immersion, the adhesive strength gradually decreased. This reduction was likely caused by continuous water penetration, which weakened interfacial hydrogen bonding between the PTA/AG hydrogel and porcine skin. Meanwhile, the gradual leaching of unincorporated TA-Na and AG molecules during long-term soaking may have further contributed to the decline in adhesion strength.

The wet adhesion strength of the PTA/AG hydrogel on porcine skin exceeded 14 kPa at 4 °C, 25 °C, and 37 °C (Figure 3e). The results indicated that the PTA/AG hydrogel maintained stable skin adhesion over a temperature range of 4–37 °C. This temperature-tolerant adhesion may be attributed to the dual-network structure, in which the PTA primary network provides basic cohesion, while the AG-derived secondary network further reinforced the network cohesion. To evaluate the wet adhesion performance of the PTA/AG hydrogel under different wet environments, adhesion tests were conducted on porcine skin in water, 1 mol/L NaCl solution, and an extreme acidic solution (pH = 2). As shown in Figure 3f, the PTA/AG hydrogel showed the highest adhesion strength (16.37 ± 1.33 kPa) in the acidic solution, whereas the PTA/AG hydrogel showed the lowest adhesion strength (5.76 ± 0.47 kPa) in 1 mol/L NaCl solution. The results indicated that the PTA/AG hydrogel exhibited good adhesion properties in water, 1 mol/L NaCl solution and an acidic solution (pH = 2). The lowest adhesion strength observed in 1 mol/L NaCl solution was likely due to the high ionic strength, which screened intermolecular interactions and weakened both network cohesion and tissue adhesion. The enhanced adhesion under acidic conditions may be mainly associated with the protonation of carboxylate groups in the PTA/AG hydrogel, particularly those derived from TA-Na and AG. This protonation may reduce electrostatic repulsion between polymer chains, leading to closer molecular packing and stronger hydrogen bonding among carboxyl and hydroxyl groups.

### 3.4. Free-Radical-Scavenging Properties of the PTA/AG Hydrogel

**Figure 4:**
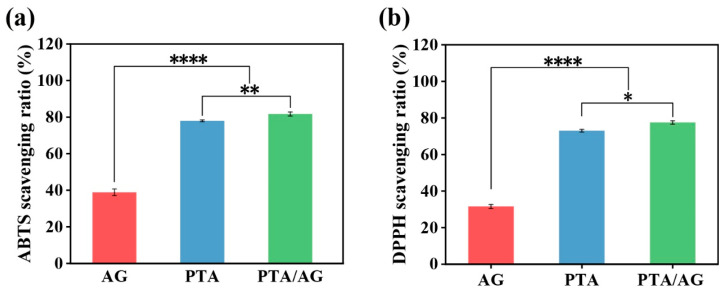
(**a**) ABTS radical scavenging of AG, PTA, and PTA/AG groups. (**b**) DPPH radical scavenging of AG, PTA, and PTA/AG groups (*n* = 3, **** *p* < 0.0001, ** *p* < 0.01 and * *p* < 0.05).

Excessive free radicals at wound sites can induce oxidative damage and impair the wound-healing microenvironment. Therefore, an ideal wound dressing should possess sustained free-radical-scavenging activity [34,35]. The free-radical-scavenging performance of the PTA/AG hydrogel was evaluated by measuring its scavenging rates against 1,1-diphenyl-2-picrylhydrazyl (DPPH) and 2,2′-azino-bis(3-ethylbenzothiazoline-6-sulfonic acid) (ABTS). As shown in Figure 4a,b, the AG hydrogel exhibited relatively low scavenging efficiencies toward both ABTS and DPPH, with values below 40%, indicating its free-radical-scavenging ability was insufficient. In contrast, the PTA hydrogel showed higher free-radical-scavenging activity than the AG hydrogel. In addition, as shown in Figure 4a,b, the PTA/AG hydrogel exhibited the highest scavenging rates against ABTS and DPPH, reaching 77.51 ± 0.96% and 81.68 ± 1.10%, respectively. The free-radical-scavenging activity of the PTA/AG hydrogel may be attributed to the synergistic contribution of two mechanisms. On the one hand, PTA can provide electrons or hydrogen atoms to DPPH• and ABTS•+ through its sulfur-containing redox-active structure, thereby reducing the free radicals [36,37]. On the other hand, AG may further enhance the overall free-radicals-scavenging performance of the hydrogel by providing complementary antioxidant activity [38].

### 3.5. Antibacterial Properties of the PTA/AG Hydrogel

**Figure 5:**
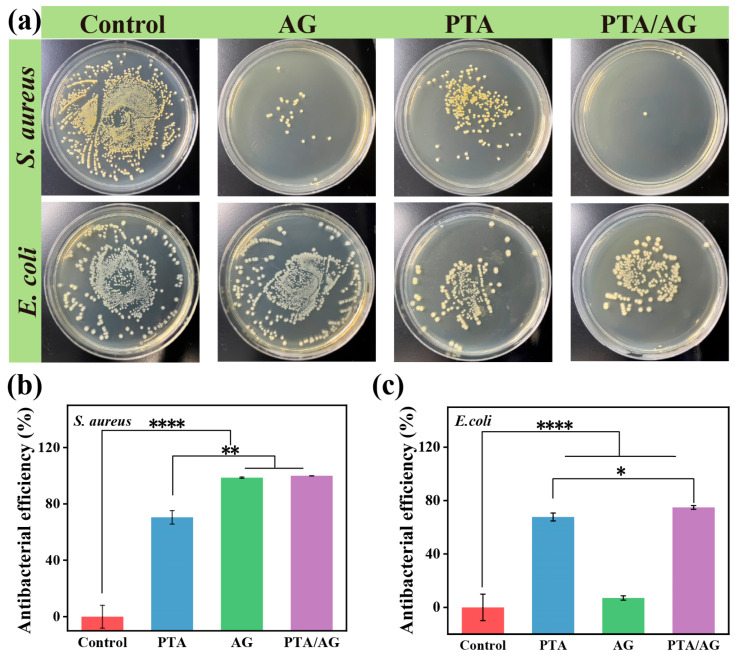
In vitro analysis of antibacterial activity. (**a**) Photos of living bacteria on agar plates after treatment with AG, PTA and PTA/AG hydrogels. (**b**,**c**) Antibacterial efficiency of AG, PTA and PTA/AG hydrogels for *S. aureus* and *E. coli* (*n* = 3, **** *p* < 0.0001, ** *p* < 0.01 and * *p* < 0.05).

Bacterial infection is a key factor contributing to delayed wound healing. Therefore, effective antibacterial activity is an important requirement for hydrogel wound dressings [39,40,41]. In this study, the antibacterial activities of AG, PTA and PTA/AG hydrogels against *Staphylococcus aureus* (*S. aureus*) and *Escherichia coli* (*E. coli*) were assessed by the spread plate method. As shown in Figure 5a, the AG hydrogel exhibited antibacterial activity against *S. aureus* (>95%) but showed only limited activity against *E. coli* (<10%) (Figure 5b,c). Furthermore, the antibacterial activity of the PTA hydrogel against both bacterial strains remained below 70%. Notably, the PTA/AG hydrogel demonstrated improved antibacterial performance, achieving antibacterial efficiencies of 99.94 ± 0.08% against *S. aureus* and 74.85 ± 1.35% against *E. coli* (Figure 5b,c).

The antibacterial activity of the PTA/AG hydrogel against *S. aureus* was mainly attributed to the antibacterial properties of AG. This enhanced activity may be attributed to the ability of AG to disrupt bacterial biofilms, interfere with key bacterial enzymes and virulence-related processes, and potentially suppress cell-wall-associated resistance proteins, such as PBP2a [14,42]. Meanwhile, the PTA/AG hydrogel also demonstrated a moderate antibacterial effect against *E. coli*. This effect may be attributed to PTA, which may exert antibacterial activity through membrane-associated damage and disruption of bacterial physiological homeostasis. As a Gram-negative bacterium, *E. coli* possesses an additional outer membrane barrier, which generally makes it less susceptible than Gram-positive bacteria, such as *S. aureus*, and may explain the weaker antibacterial effect observed against *E. coli* than against *S. aureus* [43].

### 3.6. Cytocompatibility of the PTA/AG Hydrogel

**Figure 6:**
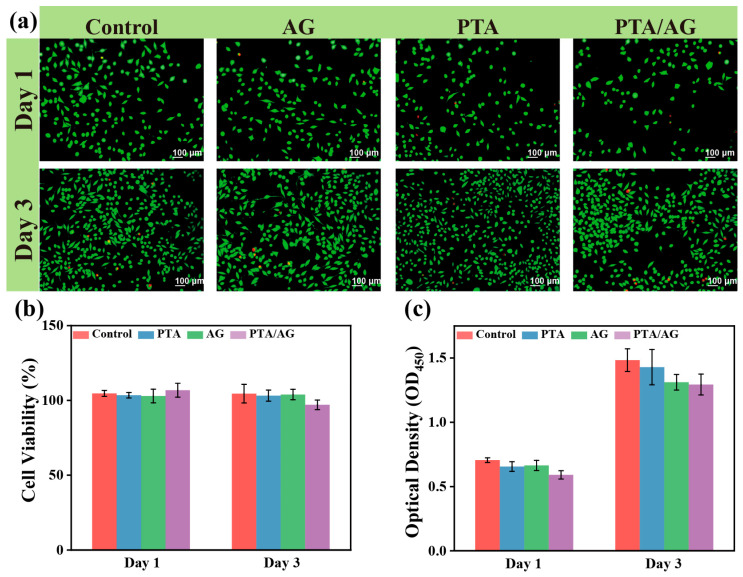
In vitro cytocompatibility analysis. (**a**) Live/dead staining of L929 cells at different times (scale bar = 100 μm); (**b**) cell viability of L929 cells after 1 and 3 days; (**c**) optical density of L929 cells at 1 and 3 days (*n* = 3).

The biological safety of hydrogels is an important prerequisite for their application in wound repair [44,45]. Calcein-AM/PI staining was used to assess the cytocompatibility of the PTA/AG hydrogel. As shown in Figure 6a, L929 cells in all experimental groups exhibited a healthy spindle-like morphology. This result indicated that AG, PTA, and PTA/AG hydrogels exhibited favorable cytocompatibility. To quantitatively evaluate cell viability after co-culture with the hydrogel samples, a CCK-8 assay was performed. As shown in Figure 6b, on both days 1 and 3, the cell viability of the PTA/AG hydrogel exceeded 95%. The results also indicated that the PTA/AG hydrogel had good cytocompatibility. Moreover, the OD values obtained from the cell viability assay showed that the PTA/AG hydrogel had no obvious adverse effect on cell viability, further suggesting favorable cytocompatibility under co-culture conditions (Figure 6c).

The favorable cytocompatibility of the PTA/AG hydrogel may be attributed to the intrinsic biocompatibility of its components. PTA is derived from TA, a naturally occurring small molecule with favorable biocompatibility, and AG is also known for its relatively low biological toxicity. Moreover, the PTA/AG hydrogel is mainly constructed through dynamic covalent interactions and physical intermolecular interactions without introducing highly toxic crosslinking agents, which may help reduce adverse effects on cell viability. These characteristics collectively contribute to the favorable cytocompatibility of the PTA/AG hydrogel and support its potential application in wound dressing.

### 3.7. ROS-Scavenging Ability of the PTA/AG Hydrogel

**Figure 7:**
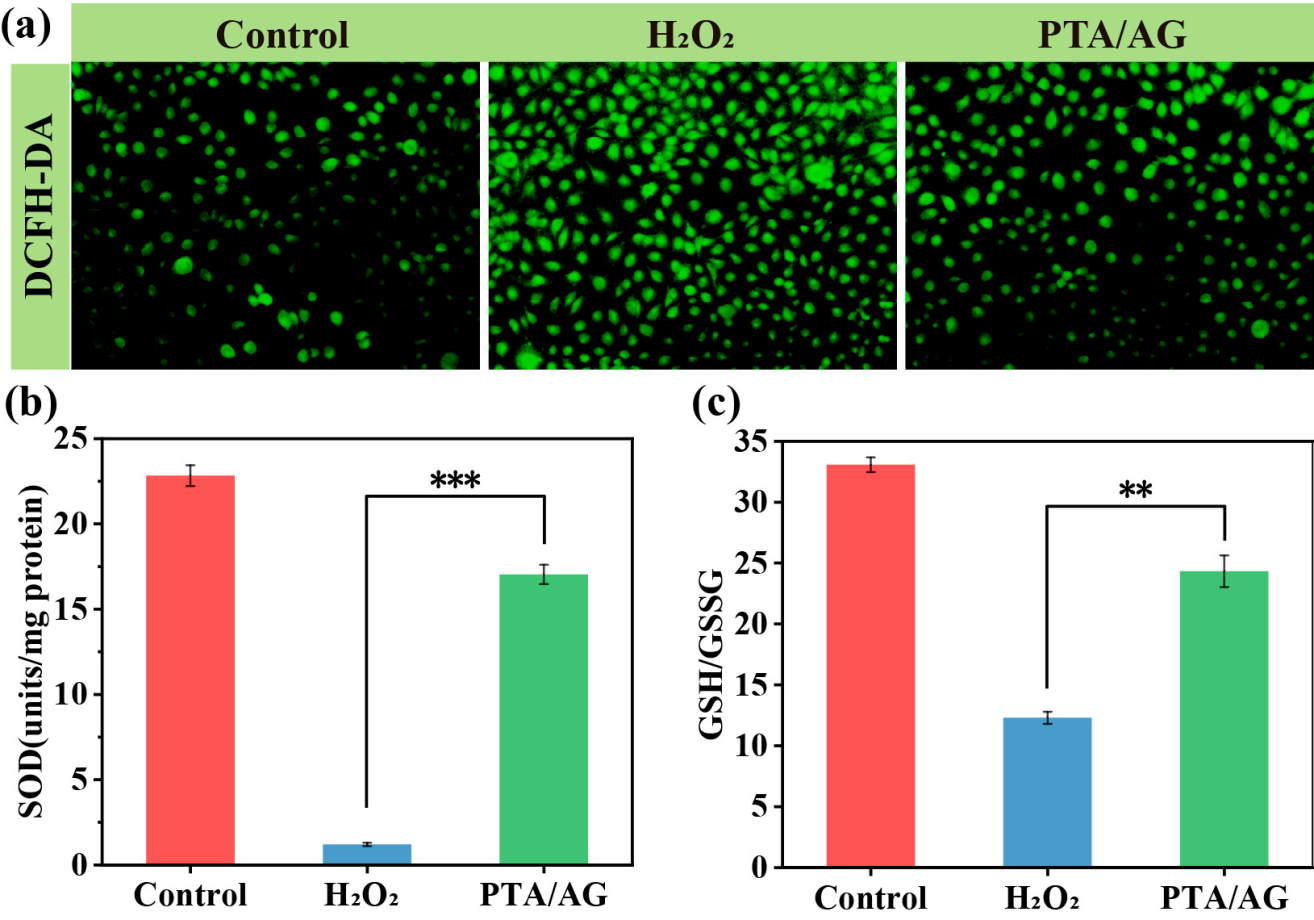
ROS-scavenging performance of PTA/AG hydrogel. (**a**) Representative images of ROS in HUVECs; intracellular levels of (**b**) SOD and (**c**) GSH/GSSG after incubation with different samples (*n* = 3, *** *p* < 0.001 and ** *p* < 0.01).

Reactive oxygen species (ROS) are involved in wound healing. However, excessive ROS accumulation can lead to oxidative stress, impair angiogenesis, and delay tissue repair [46,47,48]. In this study, the intracellular ROS-scavenging capability of the PTA/AG hydrogel was evaluated using a DCFH-DA probe. As shown in Figure 7a, a pronounced ROS fluorescence signal was observed in HUVECs after H_2_O_2_ treatment. This result indicated a significant increase in intracellular oxidative stress. In contrast, after treatment with the PTA/AG hydrogel, the intracellular ROS fluorescence intensity was markedly reduced to a level comparable to that of the control group, suggesting that the hydrogel possessed effective intracellular ROS-scavenging activity. This antioxidant effect may be attributed to the synergistic actions of AG and PTA. On the one hand, AG may enhance the activity and expression of endogenous antioxidant enzymes, such as superoxide dismutase (SOD), thereby promoting the enzymatic clearance of reactive oxygen species (ROS), including H_2_O_2_ removal [49,50]. On the other hand, the disulfide/dithiol redox pairs in PTA may quench various ROS through electron transfer and support the maintenance or regeneration of endogenous antioxidants, such as glutathione (GSH), thereby conferring cytoprotective antioxidant effects [51,52].

As a key metalloenzyme, SOD plays a crucial role in maintaining intracellular redox homeostasis [53,54,55]. As shown in Figure 7b, intracellular SOD activity after H_2_O_2_-treatment was approximately 18-fold lower than that in the control group. After subsequent treatment with the PTA/AG hydrogel, intracellular SOD activity was approximately 13-fold higher than that in the H_2_O_2_-treated group. In addition, under oxidative stress conditions, GSH can be oxidized to glutathione disulfide (GSSG), and a stable GSH/GSSG ratio is essential for maintaining normal cellular metabolism and redox balance [56,57]. As shown in Figure 7c, compared with the control group, H_2_O_2_ treatment reduced the intracellular GSH/GSSG ratio by 62.8%. Subsequent incubation with the PTA/AG hydrogel increased the GSH/GSSG ratio by 97.83% relative to the H_2_O_2_-treated group. These results indicated that the PTA/AG hydrogel had considerable potential to alleviate the oxidative stress microenvironment of wounds.

### 3.8. Degradation Performance of PTA/AG Hydrogel

**Figure 8:**
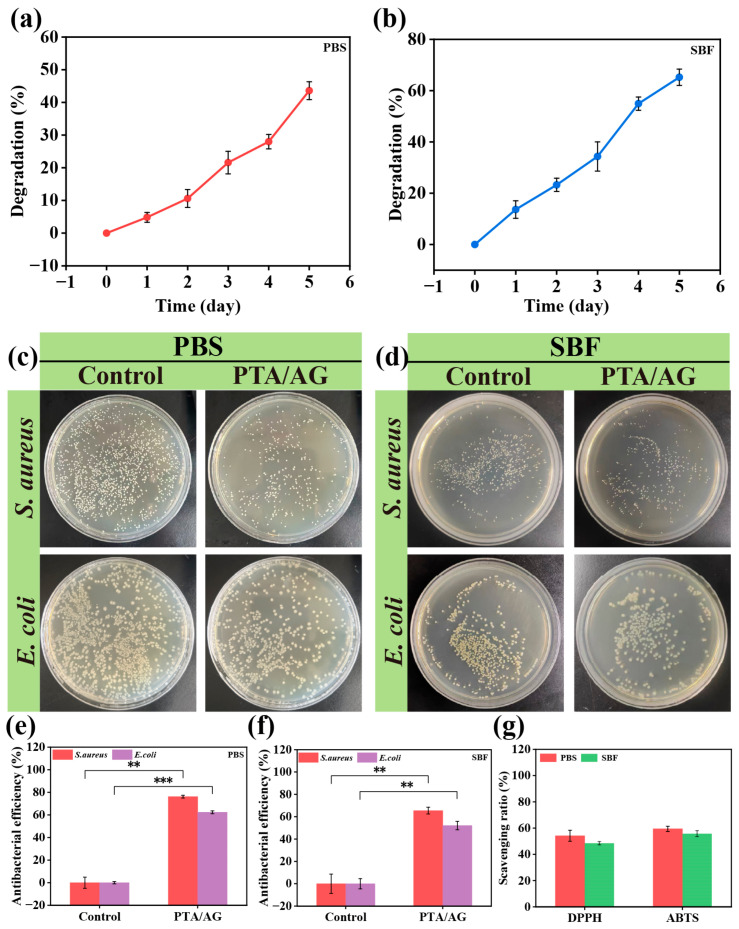
Degradation rate curves of the PTA/AG hydrogel in (**a**) PBS and (**b**) simulated body fluid (SBF). After the PTA/AG hydrogel was immersed in PBS and SBF for 5 days, respectively. (**c**,**d**) Images of living bacteria on agar plates. (**e**,**f**) Antibacterial efficiency of the PTA/AG hydrogels for *S. aureus* and *E. coli* (**g**) The ABTS and DPPH radical scavenging rates of the PTA/AG hydrogel (*n* = 3, *** *p* < 0.001 and ** *p* < 0.01).

An ideal hydrogel dressing should provide structural support and therapeutic functions, while exhibiting controllable degradation behavior that matches the tissue repair process. This performance helps create space for new tissue ingrowth and gradual material replacement during wound remodeling [58]. As shown in Figure 8a, the mass of the PTA/AG hydrogel in PBS continuously decreased with prolonged immersion time, showing a relatively stable time-dependent degradation profile. Quantitative analysis showed that the degradation rate reached 43.60 ± 2.74% after 5 days of immersion. This result may be attributed to reversible non-covalent interactions within the PTA/AG hydrogel network, including hydrogen bonding, hydrophobic association, and electrostatic interactions, which gradually weakened under long-term hydration conditions, thereby leading to the gradual disintegration of the network [59]. In addition, the ionic environment of PBS may promote the release of incompletely bound small molecules or oligomers from the hydrogel network. Notably, the degradation rate of the hydrogel was markedly accelerated in simulated body fluid (SBF), reaching 65.23 ± 3.17% after 5 days (Figure 8b). Compared with PBS, SBF had a more complex ionic composition and higher ionic strength, and it more closely resembled the in vivo environment. As a result, SBF may have more effectively weakened the non-covalent interactions between polymer chains and accelerated the relaxation and dissociation of the crosslinked network. Overall, the PTA/AG hydrogel exhibited quantifiable degradation behavior under different wet conditions, with a faster degradation rate in SBF, which more closely resembles the physiological environment. Such moderate degradation may be beneficial for tissue ingrowth and nutrient exchange during wound repair. These results indicated that the PTA/AG hydrogel has promising potential as a wound dressing for promoting tissue regeneration.

**Figure A1:**
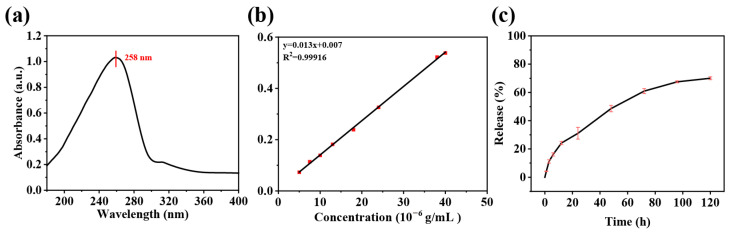
(**a**) The UV–vis spectra of AG and (**b**) the calibration curve of AG in water. (**c**) Release curve of AG in PBS at 37 °C in vitro.

To identify the component released from the PTA/AG hydrogel, the release behavior of AG in PBS was analyzed by UV–vis spectroscopy. As shown in Figure A1a, AG showed a characteristic absorption peak at 258 nm, and a calibration curve with good linearity was established in aqueous solution (y = 0.013x + 0.007, R^2^ = 0.99916) (Figure A1b). As shown in Figure A1c, the cumulative release profile showed that AG was gradually released from the PTA/AG hydrogel at 37 °C, reaching 69.37 ± 1.27% after 120 h. This result suggested that the AG could partially diffuse out of the hydrogel network during incubation, whereas the PTA network mainly remained as the hydrogel matrix. Therefore, the mass loss observed during the degradation test may be partly attributed to AG release and the gradual weakening of the physically associated dual-network structure.

Subsequently, the antibacterial activity of the immersed hydrogels was evaluated by the spread plate method. As shown in Figure 8c,d, the images showed the living bacteria on agar plates of the hydrogels after degradation for 5 days in PBS and SBF, respectively. After 5 days of immersion in PBS, as shown in Figure 8e, the antibacterial efficiencies of the PTA/AG hydrogel against *S. aureus* and *E. coli* were 76.16 ± 1.23% and 62.34 ± 1.20%, respectively. After 5 days of immersion in SBF, as shown in Figure 8f, the antibacterial efficiencies against *S. aureus* and *E. coli* were 65.45 ± 3.05% and 52.09 ± 3.78%, respectively. These results indicated that the PTA/AG hydrogel still maintained moderate antibacterial activity after 5 days of immersion. The decreased antibacterial activity may be attributed to the gradual leaching of antibacterial components, such as AG, TA, and TA-Na.

Finally, the free-radical-scavenging activity of the PTA/AG hydrogel after 5 days of immersion was evaluated by measuring its scavenging rates against DPPH and ABTS. As shown in Figure 8g, the hydrogel immersed in PBS exhibited ABTS and DPPH scavenging rates of 59.45 ± 1.98% and 54.18 ± 4.20%, respectively, whereas the hydrogel immersed in SBF showed corresponding scavenging rates of 55.76 ± 2.23% and 48.53 ± 1.24%, respectively. These results indicated that the PTA/AG hydrogel still maintained moderate antioxidant activity after 5 days of immersion. The reduced antioxidant activity may be attributed to the gradual leaching of antioxidant components, such as TA, TA-Na, AG, and soluble PTA oligomers, during immersion.

### 3.9. Limitations and Future Perspectives

Although the PTA/AG hydrogel exhibited favorable wet adhesion, antibacterial activity, cytocompatibility, antioxidant performance, and degradation behavior in vitro, the absence of in vivo wound-healing experiments remains a limitation of this study. The complex wound microenvironment, including tissue exudation, bacterial infection, inflammation, angiogenesis, and tissue remodeling, cannot be fully reproduced by current in vitro assays.

In future studies, keratinocyte migration, fibroblast scratch assays, endothelial tube formation, and macrophage inflammatory response/polarization analyses will be performed to further clarify the wound repair mechanism of the PTA/AG hydrogel. Moreover, full-thickness and infected wound models will be established to evaluate wound closure, antibacterial efficacy, inflammatory regulation, angiogenesis, collagen deposition, tissue regeneration, and long-term biosafety.

## 4. Conclusions

In conclusion, a dual-network PTA/AG hydrogel was successfully prepared. The resulting PTA/AG hydrogel exhibited favorable mechanical strength, good wet tissue adhesion, effective antibacterial activity, favorable cytocompatibility, and antioxidant properties. In addition, the PTA/AG hydrogel alleviated intracellular oxidative stress through a dual mechanism of direct ROS scavenging and reinforcement of endogenous antioxidant defenses. These findings suggest that the PTA/AG hydrogel has great potential for further development as a multifunctional wound dressing.

## Data Availability

The raw data supporting the conclusions of this article will be made available by the authors on request.

## Appendix A

### Appendix A.1. Proton Nuclear Magnetic Resonance (^1^H NMR) Spectroscopy

The ^1^H NMR spectrum of TA and PTA/AG structures were obtained in C_3_D_6_O (acetone -d6) using a INOVA 600 MHz NMR spectrometer.

### Appendix A.2. In Vitro Release Analysis of AG from PTA/AG Hydrogel

PTA/AG hydrogel samples with a known AG content were immersed in PBS solution and incubated at 37 °C. At predetermined time intervals (0, 6, 12, 24, 48, 72, 96, 120 h), an aliquot of the release medium was collected for UV–vis analysis, and the same volume of fresh PBS was added to maintain a constant release volume.

To establish the calibration curve, AG standard solutions with different concentrations were prepared in aqueous solution/PBS. The UV–vis spectrum of AG was first recorded from 200 to 400 nm, and the absorbance at 258 nm was selected for quantitative analysis. The absorbance values of AG standard solutions were plotted against the corresponding concentrations to obtain the linear calibration equation:

A=0.013C+0.007

where A is the absorbance at 258 nm and C is the AG concentration. The calibration curve showed good linearity with $R^{2}=0.99916$. The AG concentration in the collected release medium was then calculated according to this calibration curve.
